# Rodent Models of Nonalcoholic Fatty Liver Disease/Nonalcoholic Steatohepatitis

**DOI:** 10.3390/ijms141121833

**Published:** 2013-11-04

**Authors:** Kento Imajo, Masato Yoneda, Takaomi Kessoku, Yuji Ogawa, Shin Maeda, Yoshio Sumida, Hideyuki Hyogo, Yuichiro Eguchi, Koichiro Wada, Atsushi Nakajima

**Affiliations:** 1 Department of Gastroenterology, Yokohama City University Graduate School of Medicine, 3-9 Fuku-ura, Yokohama 236-0004, Japan; E-Mails: kento318@yokohama-cu.ac.jp (K.I.); yoneda@med.yokohama-cu.ac.jp (M.Y.); takaomi-kesso@hotmail.co.jp (T.K.); yuji.ogawa01@gmail.com (Y.O.); smaeda@med.yokohama-cu.ac.jp (S.M.); 2 Department of Gastroenterology and Hepatology, Kyoto Prefectural University of Medicine, Kamigyo-ku, Kyoto 602-8566, Japan; E-Mail: sumida@koto.kpu-m.ac.jp; 3 Department of Medicine and Molecular Science, Graduate School of Biomedical Sciences, Hiroshima University, Hiroshima 734-8551, Japan; E-Mail: hidehyogo@hiroshima-u.ac.jp; 4 Department of Internal Medicine, Saga Medical School, Saga University, Saga 849-8501, Japan; E-Mail: eguchiyu@me.com; 5 Department of Pharmacology, Osaka University Graduate School of Dentistry, 1-8 Yamada-oka, Suita 565-0871, Japan; E-Mail: kwada@dent.osaka-u.ac.jp

**Keywords:** NAFLD, NASH, animal model, rodent model

## Abstract

Research in nonalcoholic fatty liver disease (NAFLD), including nonalcoholic steatohepatitis (NASH), has been limited by the availability of suitable models for this disease. A number of rodent models have been described in which the relevant liver pathology develops in an appropriate metabolic context. These models are promising tools for researchers investigating one of the key issues of NASH: not so much why steatosis occurs, but what causes the transition from simple steatosis to the inflammatory, progressive fibrosing condition of steatohepatitis. The different rodent models can be classified into two large groups. The first includes models in which the disease is acquired after dietary or pharmacological manipulation, and the second, genetically modified models in which liver disease develops spontaneously. To date, no single rodent model has encompassed the full spectrum of human disease progression, but individual models can imitate particular characteristics of human disease. Therefore, it is important that researchers choose the appropriate rodent models. The purpose of the present review is to discuss the metabolic abnormalities present in the currently available rodent models of NAFLD, summarizing the strengths and weaknesses of the established models and the key findings that have furthered our understanding of the disease’s pathogenesis.

## 1. Introduction

**Figure 1:**
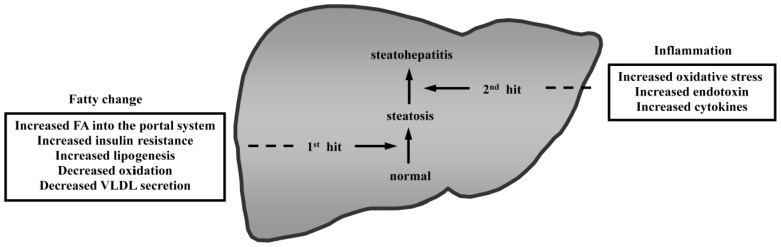
The “two-hit” theory for the pathogenesis of nonalcoholic fatty liver disease and nonalcoholic steatohepatitis. Hepatic steatosis is the initial step that occurs commonly. Then, the liver progresses from steatosis to steatohepatitis through the action of a second step. The progression from normal liver to steatohepatitis is a multistep process involving the development of fatty changes and hepatic inflammation.

In recent decades, diabetes and metabolic syndrome have become increasingly prevalent, and these conditions are strongly associated with obesity [1–4]. Accordingly, the incidence of nonalcoholic fatty liver disease (NAFLD), which is a hepatic manifestation of the metabolic syndrome, has also increased. NAFLD is a major form of chronic liver disease that is not associated with significant alcohol consumption. NAFLD is a clinical and pathological term that encompasses a disease spectrum ranging from simple steatosis to steatohepatitis, even including hepatocellular carcinoma (HCC), and is commonly observed in severely obese patients [5–10]. Nonalcoholic steatohepatitis (NASH), a type of NAFLD, is characterized by steatosis, liver inflammation, hepatocellular ballooning, and progressive liver fibrosis. NASH can ultimately lead to cirrhosis and end-stage liver disease. The development of NAFLD/NASH is determined by the interaction of genetic and environmental factors. Much research has focused on NAFLD as a complex, polygenic disease. Furthermore, the pathogenesis of NASH is thought to be a multistep process in which steatosis is the initial step and cytokines and oxidative stress induced by inflammation are important subsequent steps (Figure 1) [11]. In recent years, it has been proposed that NAFLD may actually result as a consequence of multiple parallel hits, such as gut- and adipose tissue-derived factors [12]. Clinical research into the mechanisms of NAFLD/NASH development is constrained by ethical considerations, particularly with respect to obtaining tissue samples, and by a limited ability to delineate causes and effects from amongst the complex, interactive pathogenic pathways that are involved in this condition [13]. Understandably, the development of effective preventative and therapeutic options for NASH has been hindered by the unclear etiology and pathogenetic mechanisms of this condition [14–17]. Rodent models can provide critical information toward an understanding of the molecular mechanisms responsible for this condition. Furthermore, rodent models of NAFLD/NASH are important for understanding the etiology of this disease and are valuable in the development of efficient therapies for this condition [18–20]. In recent years, several new rodent models resembling the pathogenesis of human NAFLD/NASH have been described. These models appear to be promising tools for researchers who investigate key issues in NAFLD/NASH. In this article, the currently available rodent models of NAFLD/NASH are reviewed.

## 2. Identification of Articles on Rodent Models of NAFLD/NASH

**Figure 2:**
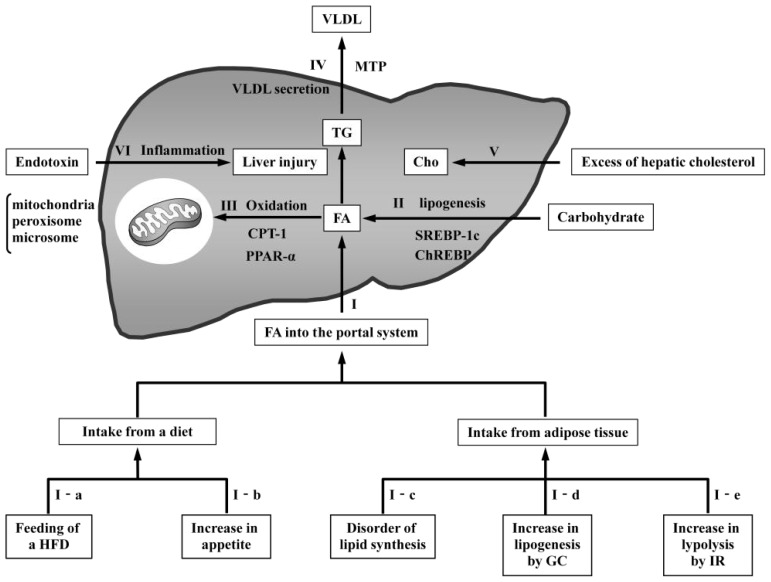
Mechanisms of NAFLD pathogenesis in rodent models. Dietary triglycerides (TG) are transported in the circulation as chylomicrons. The TG in chylomicrons are degenerated into fatty acids (FA) by lipoprotein lipase (LPL) and delivered to the liver. Furthermore, FA degenerated in adipose tissue are also delivered to the liver. (**I**) Increased FA from dietary intake or adipose tissue cause fatty changes in the liver. (**I-a**) Feeding of a high-fat diet (HFD) induces an increased supply of FA; (**I-b**) increased appetite causes an increased supply of FA as a result of excess dietary intake; (**I-c**) disordered lipid synthesis in adipose tissue places additional FA directly into the portal system; (**I-d**) high circulating glucocorticoid (GC) levels cause increased FA induced by lipogenesis; and (**I-e**) resistance to the antilipolytic action of insulin in adipose tissue results in the excessive release of FA; (**II**) Hepatic FA are synthesized from carbohydrates through pathways regulated by sterol regulatory element-binding protein-1c (SREBP-1c) and carbohydrate response element-binding protein (ChREBP). The overexpression of these proteins produces excessive FA in the liver, leading to fatty changes; (**III**) FA oxidation occurs in the mitochondria, peroxisomes, and microsomes. Carnitine palmitoyltransferase 1 (CPT1) controls the transport of acyl-CoA into the mitochondria. Peroxisome proliferator-activated receptor alpha (PPARα) is a transcription factor that regulates oxidation pathways. Reduced oxidation results in fatty changes by increasing hepatic FA; (**IV**) Hepatic TG are released from the liver as components of VLDL. Microsomal triglyceride transfer protein (MTP) regulates the synthesis of VLDL. Decreased VLDL secretion causes the accumulation of FA in the liver; (**V**) Hepatic cholesterol accumulation leads to calcium depletion and endoplasmic reticulum (ER) stress, with the activation of the unfolded protein response and ER stress-induced apoptosis; (**VI**) Low-dose endotoxin induces liver injury in HFD-induced steatosis.

Published reports on rodent models of NAFLD/NASH were retrieved using PubMed. First, the following search terms were used: (nonalcoholic or non-alcoholic or non alcoholic) and/or (fatty liver/fatty liver disease or steatosis or steatohepatitis) and (animal or rodent or mouse/mice and/or model/models); Second, articles on rodent models of NAFLD/NASH were selected and summarized in Table 1. Thirty-nine rodent models were selected after a thorough article search and classified according to the mechanisms described in the next section (Figure 2). In Table 1, the characteristics of each model are given in terms of the presence or absence of the following: increased plasma fatty acids (FA) levels, obesity, insulin resistance (IR), inflammation, fibrosis, and carcinoma. These characteristics are often observed in human NAFLD/NASH. Of the 43 selected models, 30 models exhibited increased plasma FA levels, 23 exhibited obesity, 31 exhibited IR, 21 exhibited apparent inflammation, 13 exhibited fibrosis, and six exhibited carcinoma. Only two models exhibited all six characteristics.

## 3. Classification of Rodent Models of NAFLD/NASH

NAFLD/NASH can be simply modeled in rodent animals through a wide variety of factors that lead to changes in hepatic fat disposition, causing imbalances in the import and export of FA. Alternatively, an increase in hepatic cholesterol can lead to NAFLD/NASH in rodents. In addition, endotoxin-induced liver inflammation can also cause NAFLD/NASH pathogenesis. The major approaches to modeling the induction of NAFLD/NASH in rodents can be classified as follows: (I) models with increased FA delivery to the liver; (II) models with increased *de novo* lipogenesis; (III) models with reduced oxidation; (IV) models with reduced very-low-density lipoprotein (VLDL) secretion; (V) models with increased hepatic cholesterol; and (VI) models with endotoxin-induced liver inflammation (Figure 2).

### 3.1. Models with Increased FA Delivery to the Liver

Unequivocal data support the role of circulating FA in the development of hepatic steatosis [21]. Elevated levels of circulating FA occur in patients with NAFLD, resulting in the hepatic uptake of FA [22]. Despite elevated circulating FA, hepatocyte uptake of these toxins occurs [23]. Physiological interrelationships exist between FA metabolism, IR, dyslipidemia, and TG levels in subjects with NAFLD [3,24–29]. In addition, FA inhibit insulin-stimulated glucose uptake [30,31]. Accordingly, increased FA delivery to the liver seems to be associated with NAFLD pathogenesis. As models with increased FA delivery into the liver, “Models involving feeding of a high-fat diet (HFD)”, “Models with increased appetite”, “Models with disordered lipid synthesis in adipose tissue”, “Models with increased glucocorticoids (GC)” and, “Models with increased IR”, are described below.

#### 3.1.1. Models Involving Feeding of a High-Fat Diet

After a meal with a high fat content is consumed, significant amounts of chylomicron-TG are delivered to the liver and undergo lipolysis in the lysosomes, resulting in the release of large amounts of FA. An increase in diet-derived FA results in steatosis, diabetes, and obesity [21]. These models are useful because they do not require an unphysiological procedure to produce characteristics similar to those of NASH. However, the minimal amount of fibrosis and the absence of HCC are the limitations.

##### 3.1.1.1. Mice with Chronic Exposure to a HFD

C57/BL6 mice are a particularly good model for studying human metabolic syndrome because these mice develop obesity, hyperinsulinemia, hyperglycemia, and hypertension when allowed *ad libitum* access to a HFD but remain lean and physically normal when their dietary intake is restricted to normal chow [32]. In a recent longitudinal study, the chronic administration of a HFD led to impaired glucose tolerance and the development of mild steatohepatitis in male C57Bl/6J mice [33]. However, severe fibrosis and carcinoma, which are characteristics of advanced NASH, do not appear even after prolonged exposure.

##### 3.1.1.2. Mice with Intragastric Overfeeding of a HFD

In mice with a long-term gastrostomy tube, forced overfeeding with a liquid HFD induced obesity (with a 71% larger final body weight) and IR. Of these mice, 46% developed steatohepatitis (including mild fibrosis) with increased plasma alanine aminotransferase (ALT) levels in the absence of tumor necrosis factor (TNF) signaling or cytochrome P450 (CYP) 2E1 induction [34]. However, the procedure required to produce this model is complicated.

##### 3.1.1.3. Mice Fed a Combination of Fructose and a HFD

A recently published rodent feeding model combines fructose syrup and a HFD enriched with trans and saturated fatty acids (mimicking the composition of a “fast-food” meal) and promotes physical inactivity of the rodents by removing wire racks from the animal cages [35]. These mice develop severe obesity, glucose intolerance, hyperinsulinemia, and substantial hepatic steatosis with necroinflammation over 16 weeks. In addition, diets rich in fructose can lead to oxidative stress and expression of proinflammatory cytokines through increased intestinal translocation of bacterial endotoxin, which elevates the level of endotoxin in portal blood and subsequently, causes activation of Kupffer cells in the liver [36–39]. Indeed, a shift in dietary patterns towards a sugar-rich diet is known to be risk factor for the development of NAFLD in humans [40]. However, severe fibrosis and carcinoma do not appear despite prolonged exposure.

#### 3.1.2. Models with Increased Appetite

Rodent models with increased appetite exhibit an increased supply of FA in the liver as a result of their excess in the diet, leading to metabolic syndrome including fatty liver [41–43]. These models include obesity models with known or unknown genetic abnormalities. When these models are used to study NAFLD/NASH, one must remember that the models do not reflect the effects of physiological conditions.

##### 3.1.2.1. Leptin-Deficient Mice (*ob/ob* Mice)

One of the molecules that regulate energy balance in mice is the *obese* (*ob*) gene [44–46]. *ob/ob* mice have been extensively studied and represent a naturally occurring model of NAFLD [47]. These mice are leptin-deficient because a mutation in the *ob* gene, which encodes leptin, prevents its synthesis. In the absence of leptin, *ob/ob* mice are hyperphagic, inactive, and become severely obese. These mice also exhibit severe IR and hyperinsulinemia, resulting in hyperglycemia and hyperlipidemia. Most importantly, they spontaneously develop fatty livers when fed a normal chow. However, *ob/ob* mice do not exhibit steatohepatitis, including inflammation and fibrosis, because of their leptin deficiency despite significant obesity. Furthermore, this model is different from the situation in humans, in that the plasma concentration of leptin in human NASH patients is higher than that in control subjects.

##### 3.1.2.2. Leptin-Resistant Mice (*db/db* Mice)

The well-characterized recessive mutation of *diabetes* (*db*) also results in profound and early-onset obesity [48]. Mice homozygous for the *db* mutation exhibit an obesity phenotype nearly identical to the phenotype of *ob/ob* mice. *db/db* mice have elevated serum leptin levels because of a deficiency in leptin receptor (Ob-R) [49]. These mice develop significant obesity and insulin resistance from overfeeding and are able to develop macrovesicular hepatic steatosis. However, despite significant obesity, these mice do not develop fibrosis when fed a normal chow. To develop the symptoms of steatohepatitis, including fibrosis, this model requires induction with a second hit, such as a methioninecholine-deficient (MCD) diet [50].

##### 3.1.2.3. Zucker Fatty (*fa*/*fa*) Rats

The rat gene *fatty* (*fa*) has been shown to be a homolog of the mouse *db* gene, and *fa*/*fa* rats are also thought to develop obesity and diabetes as a result of a mutation in the Ob-R gene locus [51]. These rats are similar to *db/db* mice in that they develop the symptoms of steatohepatitis upon induction with a second hit.

##### 3.1.2.4. Melanocortin 4 Receptor (MC4R)-Deficient Mice

Mice with deleted *MC4R*, which is known to regulate food intake and lipid metabolism [52], exhibit steatohepatitis and even liver fibrosis and hepatocellular carcinoma when fed a HFD [53]. Indeed, several pathogenic mutations of *MC4R* are identified at a high frequency in severe early-onset obesity and NAFLD in humans [54].

##### 3.1.2.5. Other Genetic Models with Increased Appetite

Mice in which the gene for the leptin receptor (*Lepr*) is replaced with a substitution mutant at Tyr_1138_ (*Lepr^S1138^*) through homologous targeting (LRb^S1138/1138^ knockin), mice in which the whole brain carries a Nestin-Cre allele, a floxed signal transducer and activator of the transcription-3 (*Stat3*) allele (Nestin-Cre STAT3 knockout), and mice with a neuron-specific conditional Shp2 deletion generated by crossing a pan-neuronal Cre-transgenic line (CRE3) with Shp2^flox/flox^ (CRE3/Shp2 knockout) exhibit hyperphagia, reduced energy expenditure, obesity with preserved reproductive and growth function, and steatosis without inflammation or fibrosis [55–57].

KKAy mice (also called lethal yellow KK mice) were originally developed by crossing KK mice with yellow obese mice (Ay mouse). KKAy mice (the yellow offspring obtained from a cross of black KK females with obese yellow Ay males) are obese, hyperglycemic, hyperinsulinemic, hypertriglyceridemic, hypercholesterolemic, insulin resistant, and exhibit steatosis with inflammation (steatohepatitis) because of the antagonism between melanocortin receptor 4 (MC4R) and the ectopic expression of the agouti protein [58]. The phenotype of KKAy mice, including altered adipokine expression, closely resembles metabolic syndrome in humans, indicating the potential usefulness of this strain as a model of metabolic syndrome-related NASH.

Fatty liver Shionogi (FLS)-*ob* (FLS-*ob*) mice exhibit a complex polygenic trait and develop remarkable obesity and diabetic symptoms. FLS-*ob* mice develop not only hepatic steatosis but also steatohepatitis with fibrosis. Steatosis develops more quickly in these mice than in FLS and *ob/ob* mice [59]. Moreover, FLS-*ob* mice develop HCC earlier than FLS mice.

Otsuka Long-Evans Tokushima fatty (OLETF) rats are a commonly studied model of obesity and type 2 diabetes. This strain of rats was selectively bred for the null expression of the cholecystokinin-1 receptor and thus exhibits feedback defect in satiety, resulting in hyperphagia, obesity, and IR [60]. In addition, the development of hepatic steatosis and mild inflammation is recognized in OLEFT rats. These rats develop inflammation and fibrosis when fed an MCD diet.

#### 3.1.3. Models with Disordered Lipid Synthesis in Adipose Tissue

Disordered lipid synthesis in adipose tissue increases the flow of additional FA directly into the portal system. The rate of FA release, into the systemic circulation from adipose tissue, increases directly in obese subjects with NAFLD because of the excessive gene expression of hormone-sensitive lipase (HSL) [61]. Models with disordered lipid synthesis in adipose tissue, exhibit steatosis as a result of the increase in hepatic FA, similar to the situation in human NAFLD/NASH. However, these models do not exhibit obesity because of a reduction in adipose tissue, and obesity is an indispensable characteristic of human NAFLD/NASH. Therefore, these models have the disadvantage of not being able to take the influence of adipocytokines from adipose tissue into account.

##### 3.1.3.1. aP2-nSREBP-1c-Overexpressing Mice

Sterol regulatory element-binding proteins (SREBPs) are a family of membrane-bound transcription factors that principally regulate lipid synthesis. Transgenic mice expressing nuclear SREBP-1c (nSREBP-1c) in adipose tissue, under the control of the aP2 promoter, an inherited lipodystrophic model with IR and steatosis, spontaneously developed steatohepatitis [62]. Despite a lack of obesity, nSREBP-1c transgenic mice exhibit IR, hypertriglyceridemia, elevated levels of transaminases, and mild inflammation, detected on histological liver specimens that are similar to the findings in human NASH [62]. However, fibrosis is not observed in this model.

##### 3.1.3.2. A-ZIP/F-1-Overexpressing Mice

Transgenic mice expressing a dominant-negative protein named A-ZIP/F-1, which inhibits the DNA binding and function of critical transcription factors for fat tissue development, have virtually no white adipose tissue and a significantly reduced amount of brown adipose tissue [63]. Similar to humans with lipodystrophic disorders, these mice suffer from lipoatrophic diabetes with severe IR, hyperglycemia, hyperlipidemia, hepatic steatosis without inflammation, fibrosis, and organomegaly [63].

##### 3.1.3.3. CD36-Deficient Mice

The transmembrane protein CD36 (fatty acid translocase) is an important fatty acid transporter expressed in peripheral tissues including muscle and adipose tissue. CD36 knockout mice exhibit elevated circulating FA and TG levels and hepatic IR as a result of impaired fatty acid storage. The affected mice develop steatosis with inflammation and fail to suppress hepatic gluconeogenesis [64].

##### 3.1.3.4. Other Models with Increased FA from Peripheral Adipose Tissue

aP2-diphtheria toxin transgenic mice and PPARγ (peroxisome proliferator activated receptor gamma) hypomorphic mice lack the massive adipose stores that are characteristic of obesity, although they still develop the clinical correlates of hyperlipidemia and fatty liver [65,66].

#### 3.1.4. Models with Increased Glucocorticoids

Glucocorticoids (GC) stimulate lipogenesis and inhibit lipolysis. They have the greatest effect on fat deposits in the abdomen, the nape of the neck, and the face. High circulating GC levels cause increased FA levels, steatosis, visceral obesity, IR, diabetes mellitus, dyslipidemia, hypertension, and an increased risk of cardiovascular disease. Models with increased GC exhibit a range of characteristics from steatosis to steatohepatitis, but fibrosis and HCC are not observed.

##### 3.1.4.1. 11β-HSD1-Overexpressing Mice and 7B2-Deficient Mice

11β-Hydroxysteroid dehydrogenase type 1 (11β-HSD1) catalyzes the regeneration of active GC within cells [67]. 11β-HSD1 knockout mice are protected from obesity, diabetes, and dyslipidemia. Conversely, the transgenic overexpression of 11β-HSD1 in white adipose tissue results in mice with elevated intracellular GC levels, central obesity, IR, hypertension, hyperglycemia, and dyslipidemia, producing steatosis [68]. Apparent inflammation is not observed.

Circulating adrenocorticotropic hormone (ACTH) levels are higher in B6 7B2-null mice than in wild-type mice. 7B2 knockout mice exhibit a disorder in which proinsulin or proglucagon is not converted into its active form. These mice die before four weeks of age, exhibiting hepatocyte necrosis resulting in central obesity, IR, hyperglycemia, and steatosis [69].

#### 3.1.5. Models with Increased Insulin Resistance

Insulin is known to regulate not only glucose uptake but also adipose tissue lipolysis and the suppression of hepatic glucose production [70]. Therefore, of these processes, insulin sensitivity should have the most immediate contribution to glucose homeostasis under conditions of both health and disease. Of these processes, lipolysis is the most sensitive to the action of insulin, with a >90% maximal effect well within the physiological range. In obese subjects and patients with diabetes, the insulin EC_50_ (*i.e*., the insulin concentration that produces 50% of the maximal effect) is increased two- to threefold, indicating that adipose tissue lipolysis is at least as resistant to the action of insulin as muscle and liver. While insulin-stimulated glucose disposal in adipose tissue is of little quantitative importance compared with that in muscle, the regulation of lipolysis by insulin and the subsequent release of glycerol and FA into the circulation, have major implications for glucose homeostasis. Consequently, resistance to the antilipolytic action of insulin in adipose tissue, resulting in the excessive release of FA (and glycerol), would have deleterious effects on glucose homeostasis. Models with increased IR have the drawbacks of not exhibiting inflammation, fibrosis, or HCC.

##### 3.1.5.1. Insulin I-IGF-II-Overexpressing Mice

Insulin-like growth factors (IGF)-I and II are structurally related to proinsulin and exert growth-promoting and metabolic effects. In insulin clamp studies, IGF-II transgenic mice exhibited higher rates of glucose infusion and glucose uptake and greater glycolysis than control mice, resulting in obesity, IR, and hepatic steatosis, but not inflammation [71].

##### 3.1.5.2. STAT5B-Deficient Mice

Signal transducer and activator of transcription 5 (STAT5), a latent cytoplasmic transcription factor, is known to be activated in rat liver by the male pulsatile pattern of growth hormone (GH) stimulation. This activation, which involves GH pulse-induced STAT5 tyrosine phosphorylation, followed by serine or threonine phosphorylation, induces the nuclear translocation of STAT5 and has been proposed as a key event by which the effects of GH pulses are mediated. STAT5B knockout mice are deficient in lipogenesis signals from insulin, resulting in hyperinsulinemia. These mice are present with IR, hyperglycemia, hyperlipidemia, and hepatic steatosis, but not with inflammation or fibrosis [72].

### 3.2. Models with Increased *De Novo* Lipogenesis

*De novo* lipogenesis (DNL) is the metabolic pathway responsible for the conversion of excess carbohydrates into FA, which are ultimately esterified with glycerol 3-phosphate to form TG [73]. FA can also arise from DNL in response to high-carbohydrate meals, as excess glucose is metabolized to acetyl-CoA, the major substrate for FA synthesis [74]. Malonyl-CoA, the product of acetyl-CoA carboxylase (ACC), not only is a substrate for FA synthesis but also inhibits carnitine palmitoyltransferase 1 (CPT1), which regulates long-chain FA entry into mitochondria for β-oxidation [75,76]. Hepatic DNL is regulated independently by insulin and glucose through the activation of SREBP-1c and ChREBP, which transcriptionally activate nearly all genes involved in DNL [74]. The contribution of DNL to total hepatic TG production in normal subjects is small and accounts for less than 5% of the FA incorporated into secreted VLDL-TG (1–2 g/day). However, the contribution of DNL to total hepatic TG production in subjects with NAFLD is much higher and accounts for 15% to 23% of the FA incorporated into secreted VLDL-TG. Compared with insulin-sensitive subjects, insulin-resistant subjects, with a normal hepatic TG content, had a much lower rate of muscle glycogen synthesis and a diversion of most of the ingested glucose toward hepatic DNL and hepatic TG synthesis following the consumption of a high-carbohydrate meal.

#### 3.2.1. Hepatocyte-Specific PTEN-Deficient Mice

Phosphatase and tensin homolog (PTEN) is a multifunctional phosphatase whose substrate is phosphatidylinositol-3,4,5-triphosphate (PIP3) [77,78]. PTEN is also a ubiquitously expressed tumor suppressor gene that down-regulates phosphatidylinositol 3-kinases (PI3Ks). PI3Ks are activated by a range of stimuli, including various cytokines and insulin. PIP3 activates the serine-threonine kinase protein kinase B (PKB/Akt), which is involved in anti-apoptosis, proliferation, and oncogenesis. The livers of 40-week-old PTEN knockout mice exhibit macrovesicular steatosis, ballooning hepatocytes, lobular inflammatory cell infiltration, and perisinusoidal fibrosis, which are also characteristics of human NASH. By 80 weeks of age, 100% of the mice’s livers reportedly exhibit adenomas and 66% exhibit HCC [79]. Thus, PTEN is important for the prevention of adipogenic and tumorigenic transformation, and PTEN knockout mice are a novel model for NASH and especially for HCC. Although this model exhibits IR, it does not exhibit increased FA levels and obesity, which are certainly recognized in human NASH.

#### 3.2.2. IDPc-Overexpressing Mice

Nicotinamide adenine dinucleotide phosphate (NADPH) is an essential cofactor for many enzymatic reactions, including glutathione metabolism and fat and cholesterol biosynthesis. Compared with wild-type littermates, transgenic mice overexpressing cytosolic NADP^+^-dependent isocitrate dehydrogenase (IDPc), which could be a major source of NADPH for fat synthesis, exhibit a greater body weight accompanied by hyperlipidemia, steatosis without inflammation, and obesity [80].

#### 3.2.3. ChREBP-Deficient Mice

The knockout of carbohydrate response element-binding protein (ChREBP), a major transcription factor controlling the activation of glucose-induced lipogenesis in the liver of mice, results in hyperlipidemia and hyperinsulinemia and steatosis without inflammation [81].

#### 3.2.4. Other Models of *De Novo* Lipogenesis

SREBP-1a has a longer activation domain and is much more potent than SREBP-1c in transcriptionally activating known target genes. The overexpression of nuclear SREBP-1a in transgenic mice, under the control of the PEPCK promoter (Tg SREBP-1a), causes apparent IR, massively enlarged steatohepatitis without fibrosis, and the disappearance of peripheral white adipose tissue [82].

### 3.3. Models with Reduced Oxidation

FA oxidation occurs in the mitochondria, peroxisomes and microsomes. Oxidation may proceed either from the second carbon atom adjacent to the COOH as β-oxidation or from the terminal carbon as ω-oxidation. β-Oxidation occurs in the mitochondria and the peroxisomes, whereas ω-oxidation occurs in the microsomes [83]. Carnitine palmitoyltransferase 1 (CPT1), which catalyzes the rate-limiting step of mitochondrial FA β-oxidation in skeletal muscle, is located in the outer mitochondrial membrane [84]. CPT1 controls the transport of long-chain acyl-CoA into the mitochondria, where it is allosterically inhibited by malonyl-CoA. AMP-activated protein kinase (AMPK), an enzyme up-regulated by energy deprivation, directly phosphorylates and inactivates acetyl-CoA carboxylase (ACC), thereby decreasing malonyl-CoA formation, increasing FA transport into the mitochondria as well as β-oxidation, and thus restoring the energy balance. Exercise and skeletal muscle contraction activate AMPK [84], stimulate lipid oxidation, and decrease lipid deposition in both the liver and skeletal muscle. A reduction in β-oxidation results in steatosis because of the increased accumulation of hepatic FA.

#### 3.3.1. Hepatocyte-Specific RARα Dominant-Negative Overexpressing Mice

Retinoids, most notably retinoic acids (RA), exert a wide variety of profound effects on vertebrate development, differentiation, and homeostasis. In the liver, retinoids are reportedly involved in many pathobiological conditions, including regeneration, fibrosis, and cancer. RARα dominant-negative transgenic mice develop hepatic steatohepatitis early in life, and liver tumors appear after 16 months of age, but fibrosis does not occur. Retinoic acid may be needed to produce collagen. The mechanisms responsible for hepatic steatohepatitis and tumor formation are thought to be associated with a decrease in mitochondrial fatty acid oxidation, the suppression of VLDL secretion, and the production of reactive oxygen species [85].

#### 3.3.2. AOX-Deficient Mice

Acyl-CoA oxidase 1 (AOX1) catalyzes the first and rate-limiting reaction of the peroxisomal FA β-oxidation pathway of very-long-chain fatty acids (VLCFAs). AOX knockout mice exhibit severe fatty metamorphosis of the liver, spontaneous hepatic peroxisomal proliferation, increased peroxisomal FA oxidation, and liver tumors. Thus, AOX deficiency results in inflammation, cell proliferation, and carcinogenesis in the liver [86]. However, this model apparently does not develop fibrosis despite exhibiting liver tumors.

#### 3.3.3. PPARα-Deficient Mice

Peroxisomes catalyze fatty acid β-oxidation. Mice with a homozygous knockout of PPARα (peroxisome proliferator activated receptor alpha), which is a member of the steroid/nuclear receptor superfamily and mediates the biological and toxicological effects of peroxisome proliferators, do not accumulate fat under normal feeding conditions but fail to up-regulate FA oxidation, and thus develop severe steatosis when fatty acid delivery to the liver is increased by fasting [87].

#### 3.3.4. Other Models of Reduced Oxidation

The involvement of estrogens in the regulation of carbohydrate and lipid metabolism has been robustly demonstrated by studies in genetically engineered mice, including aromatase knockout mice lacking intrinsic estrogen production. The oxidation of FA under normal physiological conditions may be orchestrated by an estrogen receptor-mediated signaling pathway. Aromatase knockout mice exhibit hyperinsulinemia and steatosis without inflammation [88].

Juvenile visceral steatosis (JVS) mice are an animal model of systemic carnitine deficiency with a genetic defect in organic cation transporter 2 (*OCTN2*). The abnormalities in this strain may be caused by the accumulation of long-chain fatty acids, which are not well metabolized as a result of their disturbed entry into mitochondria in carnitine-deficient states. These mice suffer from steatosis without inflammation, hypoglycemia, hyperammonemia, growth retardation, and cardiac hypertrophy [89].

Neonatal hepatic steatosis is a fatal condition of unknown etiology. A deficit in adenosine-dependent metabolism has been proposed as a possible causative factor. Physiologically, adenosine is efficiently metabolized to AMP by adenosine kinase (ADK), an enzyme that is highly expressed in the liver. ADK not only ensures normal adenine nucleotide levels but also is essential for the maintenance of *S*-adenosylmethionine-dependent transmethylation processes, from which adenosine, an obligatory product, has to be constantly removed. ADK knockout mice develop neonatal hepatic steatosis without inflammation, providing a powerful model for the rapid development of postnatally lethal fatty liver [90].

Cystathionine beta-synthase (CBS) is subject to complex regulation, including allosteric activation by the methyl donor *S*-adenosylmethionine (AdoMet). CBS is the rate-limiting enzyme of the transsulfuration pathway, which condenses homocysteine and serine into cystathionine. CBS knockout mice develop not only steatosis but also inflammation and fibrosis concomitant with an enhanced expression of proinflammatory cytokines [91].

Chow-fed female mice with a truncating mutation (*foz*) in the Alstrom syndrome 1 (*ALMS1*) gene, exhibit disordered appetite regulation and develop obesity, diabetes, and simple steatosis. In addition, HFD-fed *foz/foz* mice exhibit severe steatohepatitis, such as lobular inflammatory infiltrate and pericellular fibrosis, through the inhibition of PPARα-mediated peroxisomal FA oxidation via hypoadiponectinemia [92].

### 3.4. Models with Reduced VLDL Secretion

Triglycerides (TG) are bound by apolipoprotein B (ApoB) and build up into a mature lipoprotein particle that is then secreted [91]. ApoB synthesis is stimulated by elevated levels of FA and TG, as well as by microsomal triglyceride transfer protein (MTP), and is inhibited by insulin [93,94]. Thus, in the presence of an elevated FA afflux to the liver and normal/decreased insulin concentrations or IR, the secretion of mature VLDL-ApoB is expected to increase. The bulk of TG incorporated into VLDL originates from intracellular storage pools rather than from de novo synthesis [94]. The balance between the FA and insulin effects, determines whether the TG are combined with the ApoB VLDL particles and secreted or, alternatively, retained within the liver. Under the condition of IR, despite increased FA levels, TG are maintained within the liver, thus inducing steatosis. This key step may be relevant to the pathogenesis of NAFLD. In fact, the level of serum TG in secreted VLDL reportedly decreased as the MTP mRNA level decreased in human NASH patients [95].

#### 3.4.1. Mice Fed a MCD Diet

Mice fed a MCD diet are a frequently used nutritional model of NASH, that induces an elevation in aminotransferase and hepatic histological changes characterized by steatosis, focal inflammation, hepatocyte necrosis, and fibrosis [96]. Previous studies, using a choline-deficient (methionine-containing) diet, suggest that the pathogenesis of hepatic steatosis may be attributable, at least in part, to impairment in hepatic VLDL secretion. This assumption is further supported by the fact that methionine and choline are precursors to phosphatidylcholine, the main phospholipid coating VLDL particles. Thus, impaired VLDL secretion may play a role in MCD diet-induced hepatic lipid accumulation in mice. However, this model does not exhibit increased FA, obesity, or IR, which are recognized features of human NASH.

#### 3.4.2. Hepatocyte-Specific MTP-Deficient Mice

Microsomal triglyceride transfer protein (MTP) is required for the secretion of ApoB-containing lipoproteins from hepatocytes and from the absorptive enterocytes of the intestine. MTP is located within the lumen of the endoplasmic reticulum (ER), where it is assumed to transfer lipids during the assembly of lipoproteins. Hepatocyte-specific MTP knockout mice exhibit moderate hepatic steatosis without inflammation when fed a low-fat diet [97].

#### 3.4.3. PITPα-Deficient Mice

Phosphatidylinositol transfer proteins (PITPs) regulate the interface between lipid metabolism and cellular functions [98]. PITPα knockout mice develop aponecrotic spinocerebellar disease, hypoglycemia, and intestinal and hepatic steatosis without inflammation [99].

#### 3.4.4. Other Models of Reduced VLDL Secretion

Phosphatidylcholine, which is produced by the methylation of phosphatidylethanolamine using *S*-adenosylmethionine in a reaction that is catalyzed by phosphatidylethanolamine *N*-methyltransferase (PEMT), is a significant component of biomembranes and is required for the formation of the VLDL particle. PEMT knockout mice exhibit steatosis partly because VLDL cannot be exported from the liver [100]. Fibrosis does not seem to occur in this model.

Apolipoprotein E (ApoE) is a structural component of all lipoprotein particles except for LDL and serves as a high-affinity ligand for lipoprotein receptors. ApoE plays important roles in the hepatic uptake of chylomicron and VLDL remnants as well as in the hepatic secretion of VLDL [101]. ApoE knockout mice fed normal chow exhibit only steatosis but are characterized by the appearance of inflammatory cell infiltration with casein, inducing a predictable low-grade, systemic inflammation [102]. Moreover, ApoE knockout mice fed a HFD are characterized by the development of histological steatohepatitis.

### 3.5. Models with Increased Hepatic Cholesterol

Hepatic cholesterol accumulates when its dietary intake increases or its elimination decreases. The primary route of cholesterol elimination from the body is via the bile through both the direct canalicular excretion of biliary cholesterol and the conversion of hepatic cholesterol to bile acids [103]. Hepatic cholesterol accumulation leads to calcium depletion and endoplasmic reticulum (ER) stress with the activation of the unfolded protein response and ER stress-induced apoptosis. Moreover, hepatic cholesterol accumulation may reduce mitochondrial glutathione stores and could be corrected by *S*-adenosyl-l-methionine, which replenishes the mitochondrial glutathione levels [104,105]. Analyses of the lipid composition of human liver samples demonstrate a progressive increase in hepatic cholesterol content in subjects with NAFLD or with NASH [106]. CD36 and macrophage scavenger receptor 1 (MSR1), which are main scavenger receptors and important for modified cholesterol-rich lipoprotein uptake, have been shown to contribute independently to the onset of inflammation in NAFLD, by affecting intracellular cholesterol distribution [107]. These results suggest that disordered cholesterol metabolism may be associated with NAFLD.

#### 3.5.1. IL-1 Ra-Deficient Mice

Interleukin 1 (IL-1) is a pro-inflammatory cytokine that plays important roles in inflammation. However, IL-1 is also involved in peripheral energy homeostasis through endocrine mechanisms that are involved in cholesterol metabolism. IL-1 receptor antagonists (IL-1 Ra) prevent the effects of IL-1. IL-1 Ra knockout mice exhibited severe steatosis and pericellular fibrosis containing many inflammatory cells, similar to the situation in human NASH, following 20 weeks of feeding with an atherogenic diet [108].

#### 3.5.2. Rabbit Fed a High-Fat and High-Cholesterol Diet

Japanese white rabbits fed a high-fat and high-cholesterol diet may be a good model of human NASH because these animals develop obesity, hyperinsulinemia, hyperglycemia, hypertension, hepatic inflammatory cell infiltration, and even hepatic fibrosis [109].

### 3.6. Model with Endotoxin-Induced Liver Inflammation

Chronic inflammation is an important contributing factor in NASH pathogenesis [110]. As a major outer membrane constituent of gram-negative bacteria, lipopolysaccharide (LPS), also referred to as endotoxin, is considered a potent inducer of hepatic inflammation. LPS may be capable of stimulating inflammation, cytokine production, and accumulation of inflammatory cells within the liver. In fact, LPS has been reported to be the predominant cause of liver neutrophil infiltration in NASH patients [111]. In addition, several previous reports have shown that gut permeability, or small intestinal bacterial overgrowth, was observed more frequently in NASH patients than in healthy control subjects [112]. Interestingly, p53, a tumor suppressor protein that regulates the cell cycle, is reported to be involved in the molecular mechanisms of hepatocellular injury associated with NAFLD via endotoin-induced liver inflammation [113].

#### 3.6.1. Mice with Intraperitoneal Injection of Low-Dose LPS

Gut-derived bacterial endotoxin may play a role in the progression of disease from simple steatosis to steatohepatitis in NASH [114]. A previous study showed that the responsivity to low-dose LPS was enhanced in HFD-induced murine steatosis, and that low-dose LPS led to liver injury and severe hepatic fibrosis in HFD-fed mice, but not in chow-fed mice [115]. In addition, obesity-induced leptin induces the overexpression of CD14 via activation of STAT3 signaling in Kupffer cells, resulting in a hepatic hyper inflammatory response to low-dose LPS, and plays a crucial role in the progression of NASH [115]. This low-dose LPS-induced NASH model exhibits increased FA levels, obesity, IR, inflammation, and fibrosis, but not carcinoma.

## 4. Conclusions

NAFLD, including NASH, likely represents the tip of the iceberg of several complex biochemical, metabolic, and clinical conditions. Despite the well-defined morphological features of these conditions, our knowledge regarding the pathogenetic mechanisms and appropriate therapeutic approaches remains mostly lacking. Rodent models are essential tools for studies of disease pathogenesis. Ideally, rodent models should not only reproduce the established pathology of the human disease but also reenact the context within which the disease develops and progresses, as described above. However, rodent models of NAFLD/NASH have a number of problems, as described below. First, some features of true steatohepatitis such as ballooning hepatocyte degeneration, in addition to simple fatty changes and inflammatory infiltrates [5,110–113], are rarely demonstrated in rodent models of NAFLD/NASH; Second, no existing model exhibits cardiovascular disease, which is the most common cause of death among NAFLD/NASH patients; Third, models with genetic alterations may not reflect human NAFLD/NASH appropriately. Thus, no existing model reproduces the complete NAFLD/NASH phenotype as encountered in clinical practice, and many of the models differ from the human disease in all but the gross histological appearance. These inconsistencies and the lack of a reliable model for progressive fibrosing steatohepatitis have hampered research in this field. However, if each of the available rodent models described in this article are used appropriately, the pathogenesis of this condition may be clarified. To develop an ideal model of NAFLD/NASH, scientists should continue to improve on the current models and to develop novel models. In doing so, scientists should be able to facilitate the advancement of knowledge on the disease’s pathogenesis, eventually leading to a full understanding of human NAFLD/NASH and the development of effective therapies for this condition.

## Figures and Tables

**Table 1:** Current rodent models of nonalcoholic fatty liver disease/nonalcoholic steatohepatitis.

	FA	Obese	IR	Inflammation	Fibrosis	Carcinoma
**I Models with increased FA delivery into the liver**						

**I-a Models involving feeding of a HFD**						

Mice with chronic exposure to a HFD	Y	Y	Y	Y	Y	N
Mice with intragastric overfeeding of a HFD	Y	Y	Y	Y	Y	N
Mice fed a combination of fructose and a HFD	Y	Y	Y	Y	Y	N

**I-b Models with increased appetite**						

*ob/ob* mice	Y	Y	Y	N	N	N
*db/db* mice	Y	Y	Y	N	N	N
Zucker fatty (*fa*/*fa*) rat	Y	Y	Y	N	N	N
MC4R knockout mice	Y	Y	Y	Y	Y	Y
LRbS1138/1138 knockin mice	Y	Y	Y	N	N	N
Nestin-Cre STAT3 knockout mice	Y	Y	Y	N	N	N
CRE3/Shp2 knockout mice	Y	Y	Y	N	N	N
KKAy mice	Y	Y	Y	Y	Y	N
FLS-*ob* mice	Y	Y	Y	Y	Y	Y
OLEFT rat	Y	Y	Y	Y	N	N

**I-c Models with disordered lipid synthesis in adipose tissue**						

aP2-nSREBP-1c overexpressing mice	Y	N	Y	Y	N	N
A-ZIP/F-1 overexpressing mice	Y	N	Y	N	N	N
CD36 knockout mice	Y	N	N	Y	N	N
aP2-diphtheria toxin overexpressing mice	Y	N	Y	N	N	N
PPARγ hypomorphic mice	Y	N	Y	N	N	N

**I-d Models with increased GC**						

aP2-11β-HSD1 overexpressing mice	Y	Y	Y	N	N	N
7B2 knockout mice	Y	Y	Y	Y	N	N

**I-e Models with increased IR**						

insulin I-IGF-II overexpressing mice	Y	Y	Y	N	N	N
STAT5B knockout mice	Y	Y	Y	N	N	N

**II Models with increased *de novo *lipogenesis**						

Hepatocyte-specific PTEN knockout mice	N	N	Y	Y	Y	Y
IDPc overexpressing mice	Y	Y	Y	ND	N	N
ChREBP knockout mice	Y	N	Y	N	N	N
PEPCK-nSREBP1α overexpressing mice	N	N	Y	Y	N	N

**III Models with reduced oxidation**						

Hepatocyte-specific RARα dominant negative overexpressing mice	ND	N	N	Y	N	Y
AOX knockout mice	N	N	ND	Y	N	Y
PPARα knockout mice	Y	N	N	N	N	N
aromatase knockout mice	Y	Y	Y	N	N	N
JVS mice	N	N	N	N	N	N
ADK knockout mice	N	N	N	N	N	N
CBS knockout mice	Y	Y	ND	Y	Y	N
Alms1 knockout mice fed a HFD	Y	Y	Y	Y	Y	ND

**IV Models with decreased VLDL secretion**						

Mice fed a MCD diet	N	N	N	Y	Y	Y
Hepatocyte-specific MTP knockout mice	N	N	Y	N	N	N
PITPα knockout mice	N	N	N	N	N	N
PEMT knockout mice	N	N	N	Y	ND	N
ApoE knockout mice	N	N	ND	N	N	N

**V Models with increased intrahepatic cholesterol**						

IL-1 Ra knockout mice with an atherogenic diet	N	N	ND	Y	Y	N
A rabbit fed a HFD and high-cholesterol diet	Y	Y	Y	Y	Y	N

**VI Models with endotoxin-induced liver inflammation**						

Mice with intraperitoneal injection of low-dose lipopolysaccharide	Y	Y	Y	Y	Y	N

Y, yes; N, no; ND, not described; ADK, adenosine kinase; Alms1, Alstrom syndrome 1; AOX, acyl-CoA oxidase; Apo, apolipoprotein; CBS, cystathionine beta-synthase; ChREBP, carbohydrate response element-binding protein; FA, fatty acids; FLS, fatty liver Shionogi; GC, glucocorticoids; HFD, high-fat diet; HSD, hydroxysteroid dehydrogenase; IGF, insulin-like growth factor; IL-1 Ra, interleukin-1 receptor antagonist; IR, insulin resistance; JVS, juvenile visceral steatosis; LR, leptin receptor; MCD, methionime/choline-deficient; MC4R, melanocortin 4 receptor; MTP, microsomal triglyceride transfer protein; OLEFT, Otsuka Long-Evans Tokushima fatty; PEMT, phosphatidylethanolamine *N*-methyltransferase; PITP, phosphatidylinositol transfer proteins; PPAR, peroxisome proliferator-activated receptor; PTEN, phosphatase and tensin homolog; RAR, retinoic acid receptor; SREBP, sterol regulatory element-binding protein; STAT3, signal transducer and activator of transcription 3.
